# Optimizing in-store warehouse safety: A DEMATEL approach to comprehensive risk assessment

**DOI:** 10.1371/journal.pone.0317787

**Published:** 2025-02-13

**Authors:** Sayed Vahid Esmaeili, Ali Alboghobeish, Hosein Yazdani, Aysa Ghasemi Koozekonan, Mostafa Pouyakian

**Affiliations:** 1 Student Research Committee, Department of Occupational Health and Safety Engineering, School of Public Health and Safety, Shahid Beheshti University of Medical Sciences, Tehran, Iran; 2 Department of HSE, Marun Petrochemical Company, Bandar-e Mahshahr, Khuzestan, Iran; 3 Department of Occupational Health and Safety Engineering, School of Public Health and Safety, Shahid Beheshti University of Medical Sciences, Tehran, Iran; Istanbul University: Istanbul Universitesi, TÜRKIYE

## Abstract

**Introduction:**

In-store warehouses can be a dangerous place due to the storage of a high volume of diverse life goods which may raise the total risk of warehouse. The turnover of goods in this kind of warehouse is very high. Therefore, safety risk is a multi-criteria problem and risk assessment of a such dynamic place needs an accurate and simple method to use. This study was conducted to design and validation of a method for risk assessment of in-store warehouses using the DEMATEL method.

**Materials & methods:**

This cross-sectional descriptive analytical study was conducted between 2015 to 2016. First, a preliminary questionnaire was prepared by reviewing the available studies and documents. After assigning the group of experts and validating the questionnaire base on the content validity index (CVI) and content validity ratio (CVR), the weight of each of the parameters affecting the safety of the warehouse was determined. Then, the risk calculation model was developed. This model was validated using the failure modes and effects analysis (FMEA) method and Bland-Altman statistical method. Finally, to simplify the use of the developed risk assessment method, the algorithm of the model was also created.

**Results:**

The results showed that 21 factors are among the main factors affecting the safety of the in-store warehouse, among which the "igniting and explosive property of the goods" factor had the most impact and the "warehouse working hours" factor had the least impact. The results achieved from the designed model were consistent with the FMEA method.

**Conclusion:**

Based on the results, the newly designed risk assessment method can analyze the risks in the in-store warehouses faster and more accurately than the existing methods.

## 1. Introduction

Warehouses serve as the linchpin of the supply chain, playing a critical role in the receipt, storage, and distribution of goods [1]. However, the occurrence of occupational accidents in these environments, often resulting in significant economic losses and heavy casualties, has raised widespread and serious concerns [2, 3]. Many of these accidents, including slips, trips, falls, bruises, cuts, and hazards associated with hazardous materials, stem from a lack of adherence to safety protocols and the inherent risks associated with warehousing operations [4–7]. These safety concerns underscore the urgent need to improve safety protocols and risk management strategies in warehouse environments. Consequently, enhancing the safety and reliability of warehouses has become a paramount objective in safety management [8, 9].

Risk identification and accurately assessing the hazards present in warehouses is a critical factor in preventing accidents [10, 11]. Numerous studies have demonstrated that implementing occupational safety measures in buildings and warehouses, although time-consuming, costly and requires a high level of perception, can significantly reduce the incidence of accidents [10, 12, 13]. Given the complexities of warehouse environments and the diversity of hazards, a deep understanding of the factors influencing warehouse safety is essential for developing and implementing effective safety programs [14, 15].These factors can be used for risk assessment.

Traditional risk assessment methods, both two-dimensional risk assessment and three-dimensional, suffer from a fundamental flaw as the neglect of the time factor in risk variations [16]. Over time, the influence of factors acting as risk factors on the probability or severity of an accident changes significantly [17]. This impact, particularly in environments with high rates of change due to work processes, affects the final results of the risk assessment. Consequently, the results of a risk assessment at a specific point in time may become obsolete and unreliable just a few hours or days later. Therefore, for studying such dynamic environments, the requirement for a dynamic and time-based approach to risk assessment is becoming increasingly apparent.

Although dynamic risk assessment methods are widely used in process industries, their application in occupational settings has received less attention [18]. In-store warehouses, as an example of workplaces with rapid and continuous changes due to the constant exchange of goods, require innovative methods for risk assessment [19]. These environments, due to their dynamic nature, create varying levels of risk for workers that cannot be managed with traditional methods [1].

In the past few decades, study on risk analysis using multi-criteria decision-making methods (MCDM) has gradually increased [20]. Multi-criteria decision-making refers to decision-making in the presence of several conflicting criteria [21]. MCDM is applied in critical real-world scenarios such as risk assessment [22], supply chain management [23], and material selection [24]. The risk identification and evaluation process require a powerful method for analyzing criteria due to the numerous factors affecting accurate diagnosis and the implementation of decisions and control measures. Various MCDM methods are available to address decision-making challenges in complex situations. These include the hierarchical analysis process method [25], simple incremental weighting [26], preference based on similarity to ideal solution [27], data envelopment analysis [28], gray relation analysis [29], compromise ranking method [30], priority organization ranking method for enrichment evaluation [31] and multi-objective optimization based on ratio analysis [32].

The variety of goods in-store warehouses and numerous factors affecting their safety highlight the need to use MCDM to examine the relationship between these factors. The compatibility of goods and safety factors in-store warehouses when placed together can directly impact the safety level of the warehouse. Decision making trial and evaluation laboratory (DEMATEL) technique has been used to investigate the severity of impact and relationships that govern safety factors in-store warehouses. DEMATEL is an effective method that collects group knowledge, analyzes interrelationships between system factors, and visualizes this structure with a cause-and-effect diagram [33].

Identifying the critical success factors (CSF) among numerous factors to improve and promote emergency management is an example of using MCDM with the DEMATEL method. The relevant results have identified the critical success factor of emergency management according to the proposed method. Finally, out of 20 influencing factors, 5 factors were identified that can be achieved step by step to enhance the effectiveness and efficiency of emergency management [34].

Ultimately, the aim of this research is to examine risk assessment methods applicable in warehouse environments and to present a dynamic risk assessment approach, particularly focusing on the integration of multi-criteria decision-making (MCDM) techniques. By critically analyzing the literature, identifying key safety factors, and utilizing the Decision-Making Trial and Evaluation Laboratory (DEMATEL) technique to assess the interrelationships among these factors, we will provide a comprehensive framework to enhance safety measures in warehouses and consequently prevent and reduce the occurrence of occupational accidents.

### 1.1. Literature review

Multiple methods have been proposed for identifying and assessing workplace hazards in various studies [35]. Methods such as FMEA, FTA, HAZOP, PHA, and ETBA have been introduced for hazard identification, while techniques such as William Fine, 3D Melbourne, and MIL-STD-882 have been utilized for hazard assessment [36]. Although these methods often provide valuable and reliable solutions, none are without flaws and will require refinement. Furthermore, these methods, at best, reflect safety conditions at the time of data collection and do not take into account the dynamic nature of environments, particularly in warehouses [12]. Moreover, studies have highlighted shortcomings such as the lack of assessment of combined hazards or simultaneous errors in the PHA method [37], the high cost and time-consuming nature of the implementation of HAZOP, and the macro-level safety assessment in the ETBA technique [38], along with the generality of the methods and their lack of adaptation for utilizing in-store warehouses.

Specific research has focused on analyzing individual types of warehouses or examining particular hazards in a one-dimensional manner. Andrejić et al. employed the Fine-Kinney method to identify and assess risks associated with internal transportation activities and forklift hazards within warehouses [39]. Additionally, Xie et al. and Zhang et al. explored fire risk in warehouses, utilizing innovative approaches such as Bayesian networks and deep learning [40, 41]. Despite the potential advantages of these methodologies, their effectiveness is contingent upon the integration of expert evaluations and well-prepared databases. This reliance on expert input and comprehensive data underscores the complexity of accurately assessing risks in warehouse environments, highlighting the need for a multifaceted approach to risk management that combines various analytical techniques.

On the other hand, numerous studies emphasize the importance and value of integrating effective risk assessment methodologies in the logistics field, particularly within warehouse environments. For instance, Andrejić et al. and Pajic et al. employed a combination of the failure modes, effects, and criticality analysis (FMECA) alongside data envelopment analysis (DEA) [42], as well as FMEA with quality function deployment (QFD) Approach [43]. These approaches were utilized to evaluate risks and prioritize hazards throughout the transportation and distribution processes of goods in warehouses. The research highlighted the efficacy of the FMEA-QFD and FMECA-DEA methodologies in identifying potential hazards through practical applications in warehouse settings.

Tsai et al. showed that when FMEA fails to identify influencing factors, critical issues may be given a lower priority or remain unresolved [44]. To address this issue, the study combined FMEA with DEMATEL to correct FMEA’s drawbacks and enhance its effectiveness. First, FMEA was used to identify areas for improvement, and then DEMATEL was used to investigate the interactive effects and causal relationships of these areas. Finally, solutions were prioritized to address the issues. The proposed method effectively combined the advantages of FMEA and DEMATEL to identify major issues and prioritize solutions in the Chinese photovoltaic cell industry [44]. Marián Bujna et al. used the DEMATEL model to analyze the risks assessed by the FMEA method. The study investigated the application of individual methods and found that the DEMATEL model can better clarify the importance and connections between individual failures [45]. An analysis of data obtained from 78 warehouse managers and 1,033 warehouse employees, indicates that hazard reduction systems (HRS) are strong predictors of safety performance, which is consistent with previous researches. Additionally, Rene B.M.’s study suggests that safety-specific transformational leadership (SSTL) is an even more important predictor of safety performance than HRS [8].

Given the literature review, the limitations of various studies widely focus on three key aspects: 1) the lack of methodological focus on the comprehensive risk assessment of in-store warehouses, 2) the inability to aggregate expert opinions in simultaneous risk assessment, and 3) the failure to provide reliable and holistic results in risk assessments using a single method. This study can incorporate the optimization method for supply chain network design under the threat of failure [46], the novel risk probability assessment and risk evaluation method for logistics warehouses [47] and modern logistics transit warehouses [48]. Moreover, a crucial evaluation of the risks associated with warehouse facilities to improve dependability and safety is discussed [2]. Moreover, the examination can derive value from the suggested risk assessment model for global construction initiatives, highlighting the interconnectedness of risks and subjective evaluations [49]. Through the integration of these discoveries, the analysis can offer a thorough examination of methodologies for risk assessment, with a specific focus on in-store warehouses, and employ the DEMATEL technique for efficient risk appraisal. As a result, the aim of this study is to provide a fast and accurate method to evaluate the safety status of in-store warehouses using the DEMATEL technique, to investigate the severity of the impact and relationships governing the factors affecting the safety of store warehouses.

As noted previously, based on the FMEA approach, the DEMATEL is the important MCDM method, which are used by this article to calculate quantitative RPN research for evaluating the risk in in-store warehouse. The objectives of this research are as follows:

- To identify and classify key risk factors in in-store warehouse environments by the relevant literature and interviewees of a case study
- To analyze the causal relationships and interdependencies and calculate the weights of critical indicators using the expert panel opinion and DEMATEL technique.
- To rank the critical risks in a warehouse facility through DEMATEL for providing decision-makers with a reference.
- To develop a dynamic risk assessment framework that adapts to temporal variations in risk factors.
- To validate the proposed framework through empirical data and comparative analysis with traditional methods (FMEA).

This research article comprises five sections. Apart from the introduction and reviews the theoretical literature in section one, the second section, explains the research methods, the third section presents the results, the fourth section is further discussions, and the last section provides conclusions to this research article.

## 2. Material & methods

### 2.1. Study design and sampling

**Fig 1:**
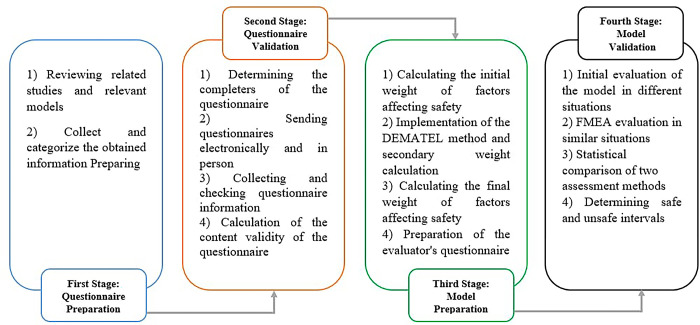
The study flow diagrams.

This descriptive-analytical study was conducted with the aim of developing a risk assessment tool for in-store warehouses. Participants in this study were recruited between 17/05/2015 and 17/9/2016. The statistical population of the study consisted of university professors with safety expertise and safety and in-store warehousing specialists. After identifying the most relevant items, the initial questionnaire, prepared in both paper and electronic versions, was sent to 21 experts. The maximum response time for the expert group members was 21 days. Fig 1 illustrates the different stages of the study, providing a brief description of each stage.

### 2.2. Questionnaire design

A literature review of studies related to the safety of in-store warehouses with main keywords as "risk assessment", "warehouse safety", "storage safety", "warehouse hazards", "storage hazards", "FMEA" and "DEMATEL" was conducted in scientific databases to examine the current methods for identifying and evaluating risks in warehouses. The studies and records of related incidents were analyzed to determine the factors affecting warehouse safety, and then the factors causing accidents were identified. In total, 110 incidents that occurred in the last two years (with and without injury) were collected from several in-store warehouses in Tehran province through consultation with warehouse management.

The analysis revealed that there were five main causes of accidents in the warehouse: product features, personnel characteristics, environmental characteristics, warehouse capacities, and warehouse output. Specifically, seven accidents were related to product features, seven were related to personnel characteristics, six were related to environmental characteristics, eight were related to warehouse capacities, and four were related to warehouse output. In total, these 32 accidents were identified as warehouse safety affecting factors.

To validate these factors and assess their severity of impact, a questionnaire was prepared consisting of 32 questions in two sections. The first section included questions related to the five factors identified in this analysis, while the second section aimed to identify other influential factors that may affect warehouse safety. The results of the questionnaire were used to develop a comprehensive risk management plan that ensures the safety of personnel and the smooth operation of the facility. By identifying the factors that contribute to accidents in the warehouse, proactive measures can take place to prevent accidents in the future.

The identified factors were determined based on the five-part Likert classification. These five parts included: not effective, very little effect, little effect, effective, and very effective. A pairwise comparison matrix was also prepared to examine the opinion of experts regarding the interaction intensity of factors if they happen at the same time in a in-store warehouse. Each item was included in the matrix both in the row and the column and the respondent assigned a code from 0 to 4 for each interaction of factors.

### 2.3. Questionnaire validation

Out of the 21 questionnaires sent, 10 were completed and collected, including 6 safety and in-store warehousing specialists, and 4 professors and faculty members of the country’s universities with safety expertise. The collected questionnaires were analyzed and the content validity index (CVI) and content validity ratio (CVR) were calculated for each factor. Due to some factors having a lower CVR value than the standard value mentioned in the sources, the number of factors decreased from 32 cases to 21 cases. To quantitatively assess content validity was used two coefficients as the CVR and CVI. To determine the CVR, experts are request to evaluate each item based on a three-part scale: "essential", "useful but not essential", and "not necessary". Then, the responses are calculated according to the following Eq (1) [50]:

$$CVR=\frac{n_{E}-\frac{N}{2}}{\frac{N}{2}}$$ (Eq 1)

where, n_E_ represents the number of experts who answered "essential", and N is the total number of experts.

To examine CVI, the method of Waltz and Bausell was used [51]. In this approach, experts specify the "relevance", "clarity", and "simplicity" of each item based on a 4-point Likert scale. Experts determine the relevance of each item from their perspective, ranging from 1 "not relevant" to 4 "highly relevant". The simplicity of the item is also specified from 1 "not simple" to 4 "highly simple", and the clarity of the item is specified from 1 "not clear" to 4 "highly clear".


CVI=Thenumberofexpertswhoratedtheitem3and4/Totalnumberofexperts (Eq 2)

The average of all three criteria for each question was also considered the average CVI and CVR values more than 0.79 and 0.62 were acceptable. Questions with a CVI of between 0.70 and 0.79 were also corrected. Finally, the average values of CVI and CVR of the remained questions were calculated.

### 2.4. Method preparation

After collecting the questionnaires, the qualitative classification of the 5-part Likert was converted to quantitative levels. The results were entered into Excel 2010 software, and the initial weight of each item was calculated. This was done by dividing the total number of scores assigned by the expert group for each section by the highest possible score of that section (score 80). The secondary weight of the factors affecting the safety of the in-store warehouse was obtained from the average of the total scores of the second part of the questionnaire in the form of a matrix of paired comparisons. The DEMATEL method was used to analyze and calculate the secondary weight after entering the scores in Excel software. Due to the large size of the obtained pairwise comparison matrix, Matrix calculator pro v5.3 software was used to calculate the inverse matrix and other matrix calculations. The final weight, that is the sum of primary and secondary weights, was calculated for each item.

### 2.5. Implementation of the DEMATEL method

DEMATEL method was first introduced by an American scientist during the Science and Humanities program between 1972 and 1976 to address complex and interrelated problems. This model is based on graph theory and enables analysis of problems using visualization methods. This structural modeling approach employs a directed and cause-effect diagram to illustrate interdependence relationships and the influencing values between factors. By analyzing the visual relationships between system elements, all elements can be categorized into a cause group and an effect group. This can help researchers better comprehend the structural relationship between system elements and identify solutions to system problems [52, 53].

DEMATEL’s method can be implemented by following these steps [54]:

1. **Generate the direct-relation matrix:** One of the methods of idea generation and group thinking among experts, such as brainstorming, thought writing Delphi, or conference, was used to determine the factors present in the problem. A list of factors presents and effective in the investigated problem was extracted from the opinion of the group of experts of the organization. In this research, the total opinions of 10 experts were formed in the matrix of paired comparisons of the questionnaire. Converting the linguistic assessments into crisp values, we obtained the direct-relation matrix A = [aij], where A is a n×n non-negative matrix, aij indicates the direct impact of factor i on factor j; and when i = j, the diagonal elements aij = 0.
2. **Determination the relationships of the governing factors by comparing their couples:** The governing relationships between the factors were determined by comparing them in pairs using the opinions of experts. The interaction intensity of factors, which represents pairwise comparisons between the factors, was determined according to the opinion of each expert.
3. **Determination of preferred numerical values:** In this step, qualitative expressions were changed to their numerical equivalents. In this research, titles such as not effective, very little effect, little effect, effective, and very effective were numbered from 0 to 4, respectively.
4. **Calculation of direct matrix using group pairwise comparisons:** The direct matrix was calculated using group pairwise comparisons. The matrices obtained from the previous step were checked and the final relationship between the two factors was decided by the majority of experts (in accepted matrices that are more consistent with each other), and the direct correlation matrix M was formed.
5. **Normalization of matrix M:** Matrix M was normalized by calculating the row sum and multiplying the inverse of the maximum by the matrix. This determined the relative intensity governing direct relationships. The normalized direct-relation matrix D = [dij] can be obtained through Eq (3). All elements in matrix D are complying with 0≤dij≤1, and all principal diagonal elements are equal to 0.
$$D=\frac{1}{Max\sum_{j=1}^{n}a_{ij}}A$$ (Eq 3)
6. **Construct the total-influence matrix:** In this step, a relative intensity matrix was formed from direct and indirect relationships. Acquire the total-relation matrix T using the Eq (4) in which I is an n×n identity matrix. The element tij indicates the indirect effects that factor i have on factor j, so the matrix T can reflect the total relationship between each pair of system factors.
$$T={D(I-D)}^{-1}$$ (Eq 4)
7. **Calculation of the possible intensity of indirect relationships:** The possible intensity of indirect relationships (of elements on each other) was calculated through the sum of geometric expansion similar to the previous one. To make the outcome more visible, we compute ri and cj through Eqs (5) and (6), respectively. The sum of row i, which is denoted as ri, represents all direct and indirect influence given by factor I to all other factors, and so ri can be called the degree of influential impact. Similarly, the sum of column j, which is denoted as cj can be called as the degree of influenced impact, since cj summarizes both direct and indirect impact received by factor j from all other factors.
$$r_{i}=\sum_{1\leq j\leq n}t_{ij}$$ (Eq 5)
$$c_{j}=\sum_{1\leq i\leq n}t_{ij}$$ (Eq 6)
  So naturally, when i = j, ri + ci shows all effects given and received by factor i. That is, ri + ci indicates both factor i’s impact on the whole system and other system factors’ impact on factor i. So, the indicator ri + ci can represent the degree of importance that factor i plays in the entire system. On the contrary, the difference of the two, ri—ci, shows the net effect that factor i has on the system. Specifically, if the value of ri—ci is positive, the factor i is a net cause, exposing net causal effect on the system. When ri—ci is negative, the factor is a net result clustered into effect group.
8. **Determination of possible hierarchy or structure of criteria:** In this step, a structure and ranking of factors were obtained by sorting them based on the values of R (influence rate), J (effectiveness rate), R+J, and R-J.

### 2.6. Expert questionnaire design

As mentioned, the characteristics of the materials and goods in the warehouse were identified as one of the factors affecting warehouse safety. To address this, it was necessary to list all the goods in the in-store warehouse and specify their characteristics. Each of the mentioned characteristics was divided into three quantitative parts based on the available sources, and three coefficients of 0.01, 0.3, and 0.69 were considered for each of these three parts according to the opinion of the research team. The coefficients were chosen based on the reasoning that the coefficient considered for the first part should be 0 due to its very low impact intensity value. However, it was not an absolute 0 because even a low impact value can have a small impact on the warehouse safety level. Similarly, the coefficient of 0.69 for a large amount of impact and the coefficient of 0.3 for an average amount of impact in the warehouse were determined. the sum of the three coefficients was equal to 1.

The risks associated with each available product were identified based on the product’s characteristics, and a final risk was determined for each product. The specified risk load was multiplied by the weight obtained from the previous step to determine the weight of the product without considering its quantity. This was done because the nature of different goods varies based on their measurement unit, and determining numerical values can create problems in developing the model. Therefore, three terms—less than the storage capacity, equal to the storage capacity, and more than the storage capacity—were used in three sections to determine the amount of goods. The desired value was marked by the form completer.

Other categories of factors that affect the safety of the in-store warehouse do not depend on the characteristics of warehouse goods. Therefore, in another section, the form completer specifies characteristics such as personnel characteristics, environmental characteristics, warehouse capacities, and warehouse output. The safety level of the warehouse is calculated by adding up the numbers assigned to each of the characteristics that affect the safety of the in-store warehouse. To simplify form completion and avoid manual calculations, a powerful software program based on Delphi programming was designed.

### 2.7. Model validation

To validate the method described, the FMEA risk identification method was used. The risks of 10 different warehouses were investigated in different conditions and times using the RPN risk assessment method. The results were presented as a percentage of the total score and compared with the results of the innovative risk assessment method using the Bland-Altman method.

### 2.8. Bland-Altman method

The Bland-Altman diagram, also known as the Tukey average difference diagram, is a statistical method used to analyze the degree of agreement between two measured parameters. It was developed by J. Martin Bland and Douglas Jel Altman and has become popular in medical statistics. The primary use of the Bland-Altman diagram is to compare two measurements that each contain some error. This chart can also be used to compare a measurement method with another method that serves as a reference standard, especially when the reference standard itself is not error-free [55, 56].

### 2.9. Ethics approval Statement and consent to participate declaration

This research was conducted in accordance with local legislation and institutional requirements. Participants gave their written informed consent to participate in this study. The consent form included elements such as introduction to the research, procedures involved, nature of participation, costs, confidentiality of information, responsibility of the researcher to answer questions, right to refuse or withdraw from the study, and acknowledgment of informed consent.

Moreover, this study was designed as a non-invasive investigation. Participants participated voluntarily and provided their findings without coercion, which generally eliminates the need for a code of ethics. However, the methodology of the study was approved by the Department of Occupational Health and Safety of Shahid Beheshti University of Medical Sciences, thus ensuring compliance with ethical standards despite the absence of a formal code of ethics. Furthermore, the opinions of participants were actively considered in the development and validation of the various phases of the study, reflecting a commitment to respect their perspectives and experiences.

## 3. Results

In this study, 21 safety and warehousing experts were invited to participate. Ultimately, 10 experts confirmed the validity of the developed tool. Individual characteristics such as expertise, age, and work experience of specialists are mentioned in Table 1.

**Table 1:** Demographic characteristics of expert group members.

N0.	Gender	Degree		Expertise	Work experience	Age
1	Male	PhD	Occupational Health	industrial Safety	23	51
2	Male			industrial Safety	21	49
3	Female			industrial Safety	13	39
4	Male			industrial Safety	4	34
5	Male	Bachelor	Management	Safety and storage	6	31
6	Male	Bachelor	Environment	Safety and storage	15	42
7	Male	Bachelor	Civil Engineering	Safety and storage	13	40
8	Male	Bachelor	Computer Science	Safety and storage	10	38
9	Male	Bachelor	Industrial Engineering	Safety and storage	8	36
10	Male	Bachelor	Mechanics	Safety and storage	18	51

### 3.1. Identification and classification of safety in-store warehouses-related factors

According to the documents, accident records, and the opinions of an expert group, it was determined that 21 factors influence the safety of in-store warehouses (Table 2). Although the intensity and degree of impact of each factor vary, these items are contributing factors to accidents in in-store warehouses.

**Table 2:** Factors affecting warehouse safety.

Parameter	Description	classification
Fire and explosive property	This category of factors relates to the inherent characteristics of the goods in the warehouse. The specific risks of each product are related to one of the mentioned factors.	Product characteristics
Chemical properties		
Weight		
Sharpness and winning		
Bad character		
Slippery property		
Number of Staff	Factors related to the working conditions of warehouse personnel are included in this category.	Personal characteristics
Moral conditions of personnel		
Shift work		
The presence of the inspector		
Training		
Assistive devices available	Environmental characteristics refer to factors that depend on the warehouse environment and the conditions and facilities available within it.	Environmental characteristics
Control and protection devices		
Cold and hot weather		
Ventilation system		
Lightening system		
Warehouse ceiling height	This category includes factors that relate to the amount of warehouse capacity.	Warehouse capacities
The total volume of the warehouse		
The number of floors per shelf		
The width of the corridors		
Season and month	This category includes factors that depend on changes in the goods stored in the warehouse.	Warehouse output

In the prepared questionnaires, two items—CVI and CVR—were calculated (See S1 Table), and their results were estimated as described in Table 3. The questionnaire was screened with 32 questions initially which was reduced to 21 questions after estimating the CVI and CVR. This was due to the lower value obtained for the CVR of the 21 questions, which resulted in their removal.

**Table 3:** Content validity of entire questionnaire.

Title	CVI	CVR
Validity of the whole questionnaire	0.95	0.74
Standard value	0.79	0.62

The weight of each factor that affects the safety of an in-store warehouse was determined in two parts: the average opinion of the expert group and the DEMATEL calculation method (Table 4).

**Table 4:** Total coefficients of factors affecting in-store warehouse safety.

No.	Parameter	Weight 1	Weight 2	Total weight
1	Fire and explosive property	0.98	0.196	1.17
2	Chemical properties	0.93	0.242	1.17
3	Weight	0.75	0.082	0.83
4	Sharpness and winning	0.80	0.190	0.99
5	Bad character	0.80	0.158	0.96
6	Slippery property	0.58	0.136	0.71
7	Number of Staff	0.73	0.166	0.89
8	Moral conditions of personnel	0.53	0.232	0.76
9	Shift work	0.70	0.173	0.87
10	The presence of the inspector	0.78	0.256	1.03
11	Training	0.75	0.302	1.05
12	Assistive devices available	0.93	0.189	1.11
13	Control and protection devices	0.93	0.177	1.13
14	Cold and hot weather	0.73	0.187	0.91
15	Ventilation system	0.70	0.170	0.87
16	Lightening system	0.83	0.129	0.96
17	Warehouse ceiling height	0.85	0.106	0.96
18	The total volume of the warehouse	0.80	0.141	0.94
19	The number of floors per shelf	0.73	0.050	0.78
20	The width of the corridors	0.83	0.036	0.87
21	Number of Staff	0.53	0.142	0.67

The goods in the in-store warehouse were divided into 18 categories based on the store’s classification and arrangement (Table 5), and the quantity was determined by an expert.

**Table 5:** Classification of goods in in-store warehouse.

Row	Category Name	Row	Category Name
1	Beverages	10	Technical
2	Cosmetic	11	Agricultural
3	Detergent	12	Vehicle Equipment
4	Dehumidifiers	13	Travel Equipment
5	Sanitary	14	Home Appliances
6	Medical	15	Sport Equipment
7	retail	16	Stationery
8	Childish	17	Heavy Household Appliances
9	Household Utensils	18	Textiles

The number of products available at the time of the study was 371, regardless of the brand and manufacturing companies. To validate the innovative method, the RPN risk assessment method was used with safety situation limits ranging from 0 to 125. The safe range was between 0 and 50, the dangerous range was between 51 and 100, and the unsafe range was between 101 and 125. After calculating the minimum and maximum innovative risk assessment scores for the entire warehouse, a comparison was made between the scores obtained from the innovative method and the FMEA method by calculating their percentage ratio. The relevant results are presented in Table 6.

**Table 6:** Results of assessments and comparisons of risk assessment methods.

No.	Evaluation time	Evaluation conditions	Place of assessment	Store’s name	FMEA score	Ratio	Limits of the FMEA method	Innovative method score	Ratio	Limits of the Innovative Method
1	Saturday	During the special sale of paper towels	FMCG Warehouse	Aram-Hyperstar	48.0	38.4	0–125	23.05	51.23	0.64–44.38
2	Sunday	During the TV special sale	HHH Warehouse	Aram-Hyperstar	8.0	8.4	0–125	12.56	43.59	0.41–28.28
3	Saturday	In normal time	LHH Warehouse	Aram-Hyperstar	8.0	8.4	0–125	19.35	40.28	0.68–47.08
4	Saturday	In normal time	TXT Warehouse	Aram-Hyperstar	8.0	4.4	0–125	5.71	50.96	0.16–11.05
5	Saturday	During the special sales of paper towels and televisions	The whole warehouse	Aram-Hyperstar	4.0	9.2	0–125	41.89	40.12	1.4–102.19
6	Tuesday	Early reopening	MCG Warehouse	Lavasan-Hyperstar	6.0	8.8	0–125	17.62	38.82	0.64–34.48
7	Wednesday	During the microwave special sale	HHH Warehouse	Aram-Hyperstar	8.0	8.4	0–125	11.68	40.43	0.41–28.28
8	Wednesday	In normal time	LHH Warehouse	Kourosh**-**Hyperstar	8.0	8.4	0–125	16.08	33.22	0.68–47.08
9	Tuesday	In normal time	TXT Warehouse	Tirajeh-Hyperstar	8.0	4.4	0–125	6.32	56.56	0.16–11.05
110	Monday	Early reopening	The whole warehouse	Lavasan-Hyperstar	7.0	1.6	0–125	39.64	37.89	1.48–102.19

According to Table 6, there is a general consensus among the data points, except for two points related to the textile warehouse. These outliers were removed due to their significant deviation from the other values. The range of difference values observed was between 1.2 and 4.5.

**Fig 2:**
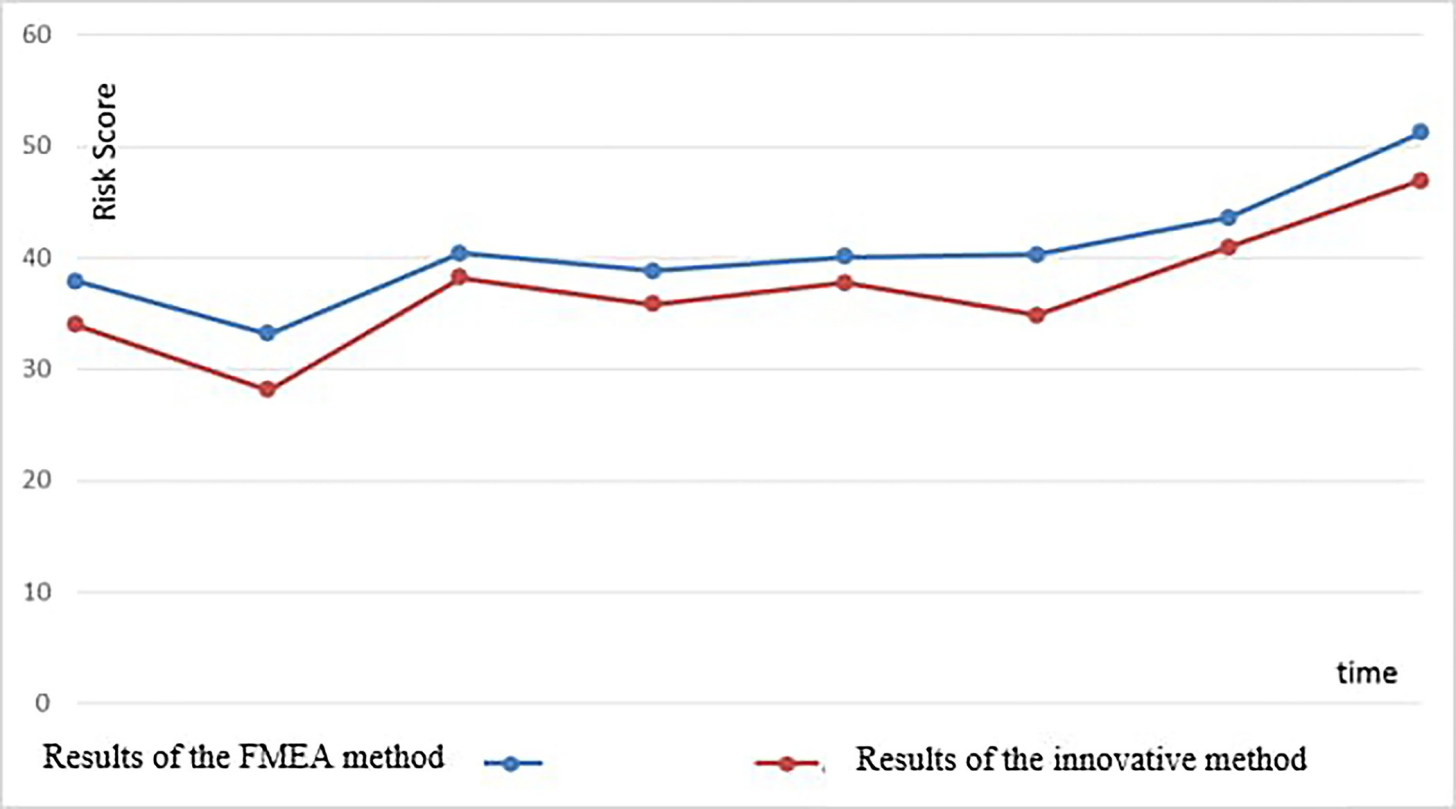
Comparison of two risk assessment methods.

In order to validate the effectiveness of the innovative method, the risk assessment results were compared to those obtained through the FMEA method. This comparison was conducted using the Bland-Altman statistical method, and the results are presented in Fig 2.

Table 7 presents the results of the innovative risk assessment method, which have been divided into three distinct intervals, each marked with a different color.

**Table 7:** Final score levels of risk.

Title	Description	Value
Acceptable risk	There is no need to implement new safety measures	41.76 >
Tolerable risk	Required safety measures, should be implemented as soon as possible	41.76–82.04
Unacceptable risk	Any action should be avoided before complete immunization	82.04 <

Table 7 provides a three-part classification system based on safety scores. The green section, representing a score of less than 41.76, indicates a relatively low-risk level where existing safety measures should be maintained. The yellow section, which ranges from 41.76 to 82.04, indicates an increased risk level, and it is recommended that measures be implemented to eliminate the causes of this risk level as soon as possible. The red section, with a score greater than 82.04, indicates a high-risk level where any action within the warehouse may result in an accident. In this case, all relevant measures should be stopped until the cause of the accident is identified and resolved. Table 8 displays the minimum and maximum amounts for each section of the store warehouse.

**Table 8:** The Minimum and maximum values of different parts of the in-store warehouse.

Minimum	Maximum	Warehouse Name	Green Area		Yellow Area		Red Area	
			Minimum	Maximum	Minimum	Maximum	Minimum	Maximum
1.48	102.19	All warehouses	0	41.7640	41.7641	82.0480	82.0481	103
0.2125	14.6668	Beverages	0	5.9942	5.9943	11.7759	11.7760	103
0.2125	14.8707	Cosmetic	0	6.0773	6.0774	11.9392	11.9393	103
0.1976	13.6359	Detergent	0	5.5729	5.5730	10.9482	10.9483	103
0.1658	11.4424	Dehumidifiers	0	4.6774	4.6765	9.1871	9.1872	103
0.2367	16.3387	Sanitary	0	6.6775	6.6776	13.1183	13.1184	103
0.1433	9.8892	Medical	0	4.0417	4.0418	7.9400	7.9401	103
0.2996	20.6756	retail	0	8.4500	8.4501	16.6004	16.6005	103
0.1512	10.4372	Childish	0	4.2654	4.2655	8.3800	8.3801	103
0.2102	14.5085	Household Utensils Technical	0	5.9295	5.9296	11.6488	11.6489	103
01829	12.6229		0	5.1589	5.1590	10.1349	10.1350	103
0.1568	10.8245	Agricultural	0	4.4239	4.4240	8.6910	8.6911	103
0.219	15.111	Vehicle Equipment	0	6.1758	6.1759	12.1326	12.1327	103
0.2171	14.9834	Travel Equipment	0	6.1236	6.1237	12.0301	12.0302	103
0.2717	13.7513	Home Appliances	0	7.6635	7.6636	15.0554	15.0555	103
0.1977	13.6448	Sport Equipment	0	5.5765	5.5766	10.9554	10.9555	103
0.1786	12.3278	Stationery	0	5.0383	5.0384	9.8980	9.8981	103
0.4098	28.2817	Heavy Household Appliances	0	11.5586	11.5587	22.7073	22.7074	103
0.1602	11.0546	Textiles	0	4.5180	4.5181	8.8757	8.8758	103

## 4. Discussion

Ensuring safety in warehouses, particularly in-store warehouses, is of paramount importance due to the inherent risks associated with these environments. While traditional risk assessment methods have proven beneficial, they often come with significant drawbacks, including time consumption and costly for employers [18, 57]. Consequently, the implementation of innovative risk assessment methodologies is essential for enhancing efficiency and effectiveness in safety management [12].

This study aimed to develop a method that can determine warehouse risk accurately and efficiently in real time. Therefore, a comprehensive risk assessment was presented based on 21 identified safety-related items in six factors as product characteristics, personal characteristics, environmental characteristics, warehouse capacities, warehouse output. To determine safety factors and to design the initial questionnaire, we employed a dual approach that combined an analysis of accident and near-miss reports documents from in-store warehouses with a thorough literature review of existing studies. This methodology ensured that our assessment was grounded in both empirical evidence and established research, allowing for a robust identification of factors that significantly influence safety within warehouse environments [58]. This eliminates the need for their re-assessment in every analysis, thereby speeding up the evaluation process. Another notable challenge in risk assessment is the reliance on documentation of accidents and near misses. Many traditional methods struggle with this aspect due to difficulties organizations face in obtaining accurate incident reports [19, 59]. For instance, the Failure Tree Analysis (FTA) method necessitates comprehensive accident documentation for effective analysis. Implementing this method without detailed statistics can be impractical [60].

After identifying and validating safety-related items, while some factors were initially believed to be more influential than others with varying degrees of intensity and significance, the present study provided evidence to support this. Based on the expert group, it was determined that some factors are more significant than others. For example, the "igniting and explosive properties" factor was deemed to be more important, while the "goods departure time" factor was considered less important. This is because accidents resulting from the explosive properties of a product are likely to be more severe than accidents caused by the product departure time [61]. Andrejić et al. identified and prioritized the most significant warehouse hazards, including fall from height, items falling from shelves during handling, forklift operations, packaging machines, electrical equipment, industrial cleaners, heaters, and forklift battery charging, potential explosion due to hydrogen gas release, acid spills [42]. Moreover, this study evaluated all products and goods in the warehouse, taking into account factors such as personnel characteristics and warehouse environmental conditions, which were not previously considered or investigated in traditional risk assessment methods.

The DEMATEL approach was utilized to calculate the weights and prioritize the identified safety items in this study. By integrating the DEMATEL approach with FMEA, we established a robust and reliable comprehensive risk assessment framework specifically designed for in-store warehouses. While past studies indicates that other decision-making approaches and FMEA had hardly been integrated to form a risk assessment [2, 43, 62], this study illustrated integration not only enhances the accuracy of risk prioritization but also facilitates a more systematic understanding of the interrelationships among various safety factors, thereby improving overall warehouse safety management. The combination of these methodologies allows for a nuanced analysis that identifies critical risk factors while considering their causal relationships, ultimately leading to more informed decision-making in safety practices [2, 63]. Such an approach is essential for developing effective strategies to mitigate risks and enhance operational safety in in-store warehouse environments [12, 33, 42, 64].

Furthermore, a review of the literature on past studies indicates that methods such as Stepwise Weight Assessment Ratio Analysis (SWARA) [65], Best-Worst Method (BWM) [2], Analytic Hierarchy Process (AHP) [12], and DEMATEL [34, 44, 45] have been employed to calculate the weights of evaluation criteria for solving MCDM problems. However, the DEMATEL method offers significant advantages compared to other MCDM approaches [53]. Unlike many other methods that assume independence between factors, this method has a high ability to identify and analyze cause-and-effect relationships among multiple factors in a complex safety system, thereby helping decision makers and managers to better understand the dependencies and interactions among these factors [66, 67]. This feature enables decision makers in fields such as safety and risk management to utilize visual maps that illustrate the relationships between factors and facilitate the identification of key and influential elements in addressing various complex problems [68].

Ultimately, this study presented a three-dimensional decision-making scale for assessing the risks of in-store environments. This scale enables precise evaluation and decision-making by assessors to adopt mitigation approaches and corrective action plans (CAP). In a study by Cebi and Ilbahar, a risk assessment method for warehouses was introduced by combining interval-valued intuitionistic fuzzy Analytical Hierarchy Process (IVIF-AHP) and a new risk assessment table, which classified the risks of transporting goods in warehouses as insignificant, marginal, or catastrophic [12].

### 4.1. Theoretical contributions

The innovative risk assessment method introduced in this study is capable of calculating the safety level of a warehouse in a relatively short time, which is a significant improvement compared to existing methods that require more time. Moreover, this method considers a wider range of factors for warehouse safety than similar methods, such as the RPN risk assessment method, which only considers three factors including severity, probability, and detection [69]. Furthermore, none of the existing methods are specifically designed for in-store warehouses risk assessment [1, 18, 40].

Another key feature of this study is the simplicity of risk assessment, as ordinary individuals can assess risk using this method with the information provided in the questionnaire, without the need for safety or warehousing experts. In addition to the mentioned features, another significant aspect of this research is the ability to assess the risk of the warehouse daily and predict safety conditions at different times of the day. The innovative risk assessment calculation method can be adapted for use in other types of warehouses by modifying input information such as available goods and products, environmental conditions, and outputs. This method can be implemented in multiple warehouses to calculate safe and unsafe intervals. Furthermore, it is very important to make the final decision in the risk assessment. the method designed in this study has a decision criterion that can help the evaluators to make the final decision.

### 4.2. Practical contributions

In rapidly changing circumstances, real-time risk assessment is of great importance [19]. Traditional risk assessment methods typically require a significant amount of time to analyze workplace conditions, and their results are often more relevant to the past [70]. In contrast, access to real-time information and the identification of hazards in warehouse environments are considered critical and vital points. A sophisticated, continuous, real-time method for dealing with quickly shifting conditions, dynamic risk analysis updates, and incorporates shifting risk levels into the overall risk profile [1]. In this regard, this study has designed and implemented a dynamic risk assessment algorithm for in-store warehouses. This algorithm enables users to assess the hazards present in warehouses with greater ease, speed, and accuracy compared to current evaluation methods. Unlike previous methods, this system takes into account all goods available in the warehouse and examines their qualitative and quantitative levels. The results of this study across various sections of in-store warehouses demonstrate the feasibility and practicality of this method as a dynamic risk assessment approach in in-store warehouse environments. Managers and safety professionals can utilize this tool for precise and timely examination of incidents in in-store warehouses. This approach not only aids in better hazard identification but also facilitates risk management.

### 4.3. Limitation

Like other studies, this study is not without its limitations. The dynamic risk assessment method presented in the research is primarily limited to the potential capabilities of the safety management in in-store warehouses. Although other studies have developed and implemented methods for risk management in high-risk warehouses, particularly chemical warehouses in various industries. Additionally, access to accurate and reliable incident reports and the gathering expert opinions under different conditions can be attributed to limitations related to data collection and analysis processes. Ultimately, although this study has been prolonged, the initiation of this research is confined to the 2015. Despite the fact that warehouse robotics is well-established in large companies and that security standards have evolved alongside new technologies in recent years, leading to changes in risk factors related to warehouse safety, the identified factors remain compatible with today’s environments due to their inherent nature. The proposed method has the capability to align with new data. This approach has particular applicability in developing countries that still rely on traditional warehousing and utilize human labor.

## 5. Conclusion

Developing a safe and productive work environment in logistics systems, particularly in in-store warehouses with a significant variety of goods, requires the establishment and reinforcement of a safety and risk management approach that raises employee awareness of the importance of safety and health protection in the workplace. This approach should be strengthened at all organizational levels, especially with the involvement of managers and supervisors who act as the primary advocates for safety. To achieve this goal, implementing a comprehensive safety program that encompasses all warehouse sections is essential. Investing in safety measures not only helps reduce accidents but also increases employee loyalty and productivity.

Risk assessment, as a key tool in safety management, assists companies in identifying and analyzing potential hazards. This article integrates the core concepts of FMEA and DEMATEL methods to enrich risk assessment approaches, which are explained in detail. Consequently, a dynamic risk assessment model was developed based on six main factors encompassing 21 items related to warehouse safety and the characteristics of the available goods. Through a specific example, risk assessment was conducted using the new approach for various sections of the in-store warehouse. The results indicated that this model could potentially aid in identifying and prioritizing hazards and ultimately serve as guidance when defining preventive-corrective actions. The main contribution of the article lies in the uniqueness of the proposed model, as the combination of methods used in this paper has not been previously employed and is intended for a distinctive environment. Moreover, by utilizing the proposed model, decision-makers can easily identify existing risks within their company, thereby creating a safer working environment for their employees.

Considering future research directions, one of the main areas will certainly be the application of the proposed model in larger samples across various real-world scenarios, as well as its application in other logistics sectors with higher risks. Furthermore, the integration of the proposed model with other decision-making and risk assessment methods to create a stronger hybrid approach is also highlighted as a promising direction for future research. Furthermore, given the development of approaches such as deep learning and computer vision in identifying and analyzing risks, the combination of this model could be proposed as a promising approach for future studies. Finally, the complexities associated with occupational health and safety (OHS) necessitate a multidisciplinary approach that includes collaboration among professionals from various fields. This approach should go beyond merely implementing protective measures and address human aspects such as the mental and physical health of employees. The selection of appropriate risk analysis methods should be based on the organizational structure and the complexity of processes.

## Supporting information

S1 Table Final data used in the study. (DOCX)

S1 Graphical abstract (TIFF)

## References

[1] Li Y, Wang H, Bai K, Chen S. Dynamic intelligent risk assessment of hazardous chemical warehouse fire based on electrostatic discharge method and improved support vector machine. Process Safety and Environmental Protection. 2021;145:425–34. 10.1016/j.psep.2020.11.012

[2] Hsu H-Y, Hwang M-H, Tsou P-H. Applications of BWM and GRA for Evaluating the Risk of Picking and Material-Handling Accidents in Warehouse Facilities. Applied Sciences. 2023;13(3):1263. 10.3390/app13031263

[3] Ren Y, Zhang J, Wang X. How does data factor utilization stimulate corporate total factor productivity: A discussion of the productivity paradox. International Review of Economics & Finance. 2024;96:103681. 10.1016/j.iref.2024.103681

[4] Rachid C, Ion V, Irina C, Mohamed B. Preserving and improving the safety and health at work: Case of Hamma Bouziane cement plant (Algeria). Safety Science. 2015;76:145–50. 10.1016/j.ssci.2015.01.014

[5] Öztürkoğlu Ö, Gue KR, Meller RD. A constructive aisle design model for unit-load warehouses with multiple pickup and deposit points. European Journal of Operational Research. 2014;236(1):382–94. 10.1016/j.ejor.2013.12.023

[6] Feyzi V, Mehdipoor S, Ghotbi Ravandi MR, Asadi M, Ghafori S. Ergonomic assessment of workstations and musculoskeletal disorders risk assessment in the central oil refinery workshop of Hormozgan province. Health and Development Journal. 2016;4(4):315–26.

[7] Hall A. Trust, uncertainty and the reporting of workplaces hazards and injuries. Health, Risk & Society. 2016;18(7–8):427–48. 10.1080/13698575.2016.1264576

[8] de Koster RB, Stam D, Balk BM. Accidents happen: The influence of safety-specific transformational leadership, safety consciousness, and hazard reducing systems on warehouse accidents. Journal of Operations management. 2011;29(7–8):753–65. 10.1016/j.jom.2011.06.005

[9] Gao J, Li X, Tao P. Occupation, risk culture, and risk perception: empirical evidence from China on COVID-19. Health, Risk & Society. 2024;26(3–4):172–200. 10.1080/13698575.2024.2333788

[10] Khajevandi AA, Shafiei Z, Zamani-Badi H, Hanani M, Esmaeili SV. Assessing the safety status of Kashan University of Medical Sciences faculties by audit method in 2018. International Archives of Health Sciences. 2021;8(3):165. 10.4103/iahs.iahs_127_20

[11] Varmazyar S, Mortazavi SB, Arghami S, Hajizadeh E. Relationship between organisational safety culture dimensions and crashes. Int J Inj Contr Saf Promot. 2016;23(1):72–8. 0. doi: 10.1080/17457300.2014.947296 25494102

[12] Cebi S, Ilbahar E. Warehouse risk assessment using interval valued intuitionistic fuzzy AHP. International Journal of the Analytic Hierarchy Process. 2018;10(2). 10.13033/ijahp.v10i2.549

[13] Lin L, Ma X, Chen C, Xu J, Huang N. Imbalanced Industrial Load Identification Based on Optimized CatBoost with Entropy Features. Journal of Electrical Engineering & Technology. 2024:1–16. 10.1007/s42835-024-01933-5

[14] Balan IL, Cioca L-I, Torretta V, Talamona L. Warehouse Threats and Loss Prevention Management in Case of Fire. Procedia Technology. 2016;22:1028–34. 10.1016/j.protcy.2016.01.138

[15] Farsani EH, Jaberi M, Jazayeri SA. Risk Perception Evaluation of Hazardous Occupational Workers in a Steel Company of Khuzestan Province. International Journal of Mechanical and Industrial Sciences (IJMIS). 2018;2(3):40–8. 10.33544/mjmie.v2i3.84

[16] DoD US. MIL-STD-882E, department of defense standard practice system safety. US Department of Defense. 2012. Access: http://everyspec.com/MIL-STD/MIL-STD-0800-0899/MIL-STD-882E_41682/

[17] Azadeh-Fard N, Schuh A, Rashedi E, Camelio JA. Risk assessment of occupational injuries using Accident Severity Grade. Safety science. 2015;76:160–7. 10.1016/j.ssci.2015.03.002

[18] Jafari MJ, Pouyakian M, khanteymoori A, Hanifi SM. Development of a framework for dynamic risk assessment of environmental impacts in chemicals warehouse using CFD-BN. International Journal of Environmental Science and Technology. 2021;18(10):3189–204. 10.1007/s13762-020-03040-0

[19] Jafari MJ, Pouyakian M, Mozaffari P, Laal F, Mohamadi H, Pour MT, et al. A new approach to chemicals warehouse risk analysis using computational fluid dynamics simulation and fuzzy Bayesian network. Heliyon. 2022;8(12). doi: 10.1016/j.heliyon.2022.e12520 36593826 PMC9803688

[20] Ilangkumaran M, Karthikeyan M, Ramachandran T, Boopathiraja M, Kirubakaran B. Risk analysis and warning rate of hot environment for foundry industry using hybrid MCDM technique. Safety science. 2015;72:133–43. 10.1016/j.ssci.2014.08.011

[21] Wang Y-M, Liu J, Elhag TM. An integrated AHP–DEA methodology for bridge risk assessment. Computers & industrial engineering. 2008;54(3):513–25. 10.1016/j.cie.2007.09.002

[22] Chen C-T, Lin C-T, Huang S-F. A fuzzy approach for supplier evaluation and selection in supply chain management. International journal of production economics. 2006;102(2):289–301. 10.1016/j.ijpe.2005.03.009

[23] Chatterjee P, Athawale VM, Chakraborty S. Selection of materials using compromise ranking and outranking methods. Materials & Design. 2009;30(10):4043–53. 10.1016/j.matdes.2009.05.016

[24] Bhushan N, Rai K. The analytic hierarchy process. Strategic decision making: applying the analytic hierarchy process: Springer London; 2004. p. 11–21. 10.1007/b97668

[25] Yeh CH. A problem‐based selection of multi‐attribute decision‐making methods. International Transactions in Operational Research. 2002;9(2):169–81. 10.1111/1475-3995.00348

[26] Charnes A, Cooper WW, Rhodes E. Measuring the efficiency of decision making units. European journal of operational research. 1978;2(6):429–44. 10.1016/0377-2217(78)90138-8

[27] Julong D. Introduction to grey system theory. The Journal of grey system. 1989;1(1):1–24.

[28] Kohl S, Schoenfelder J, Fügener A, Brunner JO. The use of Data Envelopment Analysis (DEA) in healthcare with a focus on hospitals. Health care management science. 2019;22:245–86. doi: 10.1007/s10729-018-9436-8 29478088

[29] Kuo Y, Yang T, Huang G-W. The use of grey relational analysis in solving multiple attribute decision-making problems. Computers & industrial engineering. 2008;55(1):80–93. 10.1016/j.cie.2007.12.002

[30] Juanpera M, Domenech B, Ferrer-Martí L, García-Villoria A, Pastor R. Methodology for integrated multicriteria decision-making with uncertainty: Extending the compromise ranking method for uncertain evaluation of alternatives. Fuzzy Sets and Systems. 2022;434:135–58. 10.1016/j.fss.2021.08.008

[31] Brans JP, Vincke P. Preference ranking organization method for enrichment evaluations. Management Science. 1985;31(6).

[32] Pasaribu SW, Rajagukguk E, Sitanggang M, Rahim R, Abdillah LA. Implementasi Multi-Objective Optimization On The Basis Of Ratio Analysis (MOORA) Untuk Menentukan Kualitas Buah Mangga Terbaik. JURIKOM (Jurnal Riset Komputer). 2018;5(1):50–5. 10.30865/jurikom.v5i1.571

[33] Rodrigues YG, Pinto EdO, Aquino CR, COSTA Gd, OLIVEIRA JPFGd, Campos L, et al. Occupational risk analysis in a fish warehouse: a comparative study between GUT matrix and preliminary risk analysis. Food Science and Technology. 2022;42. 10.1590/fst.28122

[34] Zhou Q, Huang W, Zhang Y. Identifying critical success factors in emergency management using a fuzzy DEMATEL method. Safety science. 2011;49(2):243–52. 10.1016/j.ssci.2010.08.005

[35] Antoniou G, Saravanou M-C, Stavrou V. An overview of risk assessment methods. 2014.

[36] Jahanvand B, Bagher Mortazavi S, Asilian Mahabadi H, Ahmadi O. Determining essential criteria for selection of risk assessment techniques in occupational health and safety: A hybrid framework of fuzzy Delphi method. Safety Science. 2023;167:106253. 10.1016/j.ssci.2023.106253

[37] Baybutt P. Requirements for improved process hazard analysis (PHA) methods. Journal of Loss Prevention in the Process Industries. 2014;32:182–91. 10.1016/J.JLP.2014.08.004

[38] Arghami S, Abbasi S, Bakhtom S, Ziaei M. Comparing of HAZOP and ETBA techniques in safety risk assessment at gasoline refinery industry. African Journal of basic & applied sciences. 2014;6(1):1–5. 10.5829/idosi.ajbas.2014.6.1.83324

[39] Pajić V, Andrejić M. Risk analysis in internal transport: An evaluation of occupational health and safety using the Fine-Kinney method. Journal of operational and strategic analytics. 2023;1(4):147–59. 10.56578/josa010401

[40] Xie J, Li J, Wang J, Jiang J, Shu C-M. Fire risk assessment in lithium-ion battery warehouse based on the Bayesian network. Process Safety and Environmental Protection. 2023;176:101–14. 10.1016/j.psep.2023.06.005

[41] Zhang Q, Tian Y, Chen J, Zhang X, Qi Z. To ensure the safety of storage: Enhancing accuracy of fire detection in warehouses with deep learning models. Process Safety and Environmental Protection. 2024;190:729–43. 10.1016/j.psep.2024.07.086

[42] Andrejić M, Pajić V. Managing warehouse risks for 3PL providers: A novel approach based on FMECA-DEA. J Organ Technol Entrep. 2024;2(2):113–21. 10.56578/jote020204

[43] Pajic V, Andrejic M, Sternad M. FMEA-QFD approach for effective risk assessment in distribution processes. J Intell Manag Decis. 2023;2(2):46–56. 10.56578/jimd020201

[44] Tsai S-B, Zhou J, Gao Y, Wang J, Li G, Zheng Y, et al. Combining FMEA with DEMATEL models to solve production process problems. PloS one. 2017;12(8):e0183634. doi: 10.1371/journal.pone.0183634 28837663 PMC5570359

[45] Bujna M, Kotus M, Matušeková E. Using the DEMATEL model for the FMEA risk analysis. System Safety: Human-Technical Facility-Environment. 2019;1(1):550–7. 10.2478/czoto-2019-0070

[46] Oshan T, Caron RJ. Optimal warehouse location and size under risk of failure. International Journal of Systems Science: Operations & Logistics. 2023;10(1):2208276. Access: https://scholar.uwindsor.ca/etd/8725

[47] Ju W, Su G, Wu L, Oforiwaa PO. The 3D-Dynamic Fire Risk Evaluation Method of Modern Logistics Warehouses: A Modified Gustav Method. Fire Technology. 2023. doi: 10.1007/s10694-023-01367-x 36776210 PMC9899158

[48] Zhang Y, Huang G. Risk Analysis of Fire Disaster in Modern Warehousing System Based on The Data of Idealized Transit Warehouse. Highlights in Science, Engineering and Technology. 2022;10:127–34. 10.54097/hset.v10i.1239

[49] Zhu F, Hu H, Xu F. Risk assessment model for international construction projects considering risk interdependence using the DEMATEL method. Plos one. 2022;17(5):e0265972. doi: 10.1371/journal.pone.0265972 35594291 PMC9122217

[50] Kishore K, Jaswal V, Kulkarni V, De D. Practical guidelines to develop and evaluate a questionnaire. Indian Dermatology Online Journal. 2021;12(2):266–75. doi: 10.4103/idoj.IDOJ_674_20 33959523 PMC8088187

[51] Waltz CF, Bausell BR. Nursing research: design statistics and computer analysis: Davis Fa; 1981.

[52] Si S-L, You X-Y, Liu H-C, Zhang P. DEMATEL technique: A systematic review of the state-of-the-art literature on methodologies and applications. Mathematical Problems in Engineering. 2018;2018:1–33. 10.1155/2018/3696457

[53] Yazdi M, Khan F, Abbassi R, Rusli R. Improved DEMATEL methodology for effective safety management decision-making. Safety science. 2020;127:104705. 10.1016/j.ssci.2020.104705

[54] Rostamzadeh S, Abouhossein A, Chalak MH, Vosoughi S, Norouzi R. An integrated DEMATEL–ANP approach for identification and prioritization of factors affecting fall from height accidents in the construction industry. International Journal of Occupational Safety and Ergonomics. 2023;29(2):474–83. doi: 10.1080/10803548.2022.2052479 35272574

[55] Mansournia MA, Waters R, Nazemipour M, Bland M, Altman DG. Bland-Altman methods for comparing methods of measurement and response to criticisms. Global Epidemiology. 2021;3:100045. eCollection 2021 Nov doi: 10.1016/j.gloepi.2020.100045 37635723 PMC10446118

[56] Haghayegh S, Kang H-A, Khoshnevis S, Smolensky MH, Diller KR. A comprehensive guideline for Bland–Altman and intra class correlation calculations to properly compare two methods of measurement and interpret findings. Physiological measurement. 2020;41(5):055012. doi: 10.1088/1361-6579/ab86d6 32252039

[57] Can GF, Toktas P. A novel fuzzy risk matrix based risk assessment approach. Kybernetes. 2018;47(9):1721–51. 10.1108/K-12-2017-0497

[58] Wasti SP, Simkhada P, van Teijlingen ER, Sathian B, Banerjee I. The Growing Importance of Mixed-Methods Research in Health. Nepal Journal of Epidemiology. 2022;12(1):1175–8. doi: 10.3126/nje.v12i1.43633 35528457 PMC9057171

[59] Fetherston T. The importance of critical incident reporting–and how to do it. Community eye health. 2015;28(90):26. 26692643 PMC4675258

[60] Omidvari M, Gharmaroudi MR. Analysis of human error in occupational accidents in the power plant industries using combining innovative FTA and meta-heuristic algorithms. Journal of Health and Safety at Work. 2015;5(3):1–12.

[61] Tubis AA, Ryczyński J, Żurek A. Risk assessment for the use of drones in warehouse operations in the first phase of introducing the service to the market. Sensors. 2021;21(20):6713. doi: 10.3390/s21206713 34695924 PMC8541576

[62] Lo H-W, Shiue W, Liou JJ, Tzeng G-H. A hybrid MCDM-based FMEA model for identification of critical failure modes in manufacturing. Soft Computing. 2020;24:15733–45. 10.1007/s00500-020-04903-x

[63] Chang X, Gao H, Li W. Discontinuous distribution of test statistics around significance thresholds in empirical accounting studies. Journal of Accounting Research. 2024. 10.1111/1475-679X.12579

[64] Zhu C. An adaptive agent decision model based on deep reinforcement learning and autonomous learning. Journal of Logistics, Informatics and Service Science. 2023;10(3):107–18. 10.33168/JLISS.2023.0309

[65] Debnath B, Bari ABMM, Haq MM, de Jesus Pacheco DA, Khan MA. An integrated stepwise weight assessment ratio analysis and weighted aggregated sum product assessment framework for sustainable supplier selection in the healthcare supply chains. Supply Chain Analytics. 2023;1:100001. 10.1016/j.sca.2022.100001

[66] Duan W, Li C. Be alert to dangers: Collapse and avoidance strategies of platform ecosystems. Journal of Business Research. 2023;162:113869. 10.1016/j.jbusres.2023.113869

[67] Huang Z, Zhou Y, Lin Y, Zhao Y. Resilience evaluation and enhancing for China’s electric vehicle supply chain in the presence of attacks: A complex network analysis approach. Computers & Industrial Engineering. 2024;195:110416. 10.1016/j.cie.2024.110416

[68] Xu X, Wei Z. Dynamic pickup and delivery problem with transshipments and LIFO constraints. Computers & Industrial Engineering. 2023;175:108835. 10.1016/j.cie.2022.108835

[69] Ghadage YD, Narkhede BE, Raut RD. Risk management of innovative projects using FMEA; a case study. International Journal of Business Excellence. 2020;20(1):70–97. 10.1504/IJBEX.2020.104841

[70] Raveendran A, Renjith VR, Madhu G. A comprehensive review on dynamic risk analysis methodologies. Journal of Loss Prevention in the Process Industries. 2022;76:104734. 10.1016/j.jlp.2022.104734

