# Supplementary material for: Optimizing in-store warehouse safety: A DEMATEL approach to comprehensive risk assessment
Source: PLoS One. 2025 Feb 13;20(2):e0317787. doi: 10.1371/journal.pone.0317787 (PMC11825006; doi:10.1371/journal.pone.0317787)
Supplement: S1 Table — (DOCX) [file pone.0317787.s001.docx]

**Table S1.** Questionnaire validity results

| Factor | Item | CVR | CVI | Decision |
| --- | --- | --- | --- | --- |
|  |  |  |  |  |
| Product characteristics | Fire and explosive property | 1 | 0.87 | Accept |
|  | Chemical properties | 1 | 0.93 | Accept |
|  | Weight | 0.8 | 0.90 | Accept |
|  | Sharpness and winning | 0.8 | 0.97 | Accept |
|  | Bad character | 0.8 | 0.93 | Accept |
|  | Slippery property | 0.8 | 0.93 | Accept |
|  | Brittleness | 0.6 | 0.97 | Reject |
| Personal characteristics | Number of Staff | 0.8 | 0.97 | Accept |
|  | Moral conditions of personnel | 0.8 | 0.9 | Accept |
|  | Shift work | 0.8 | 0.97 | Accept |
|  | The presence of the inspector | 0.8 | 0.9 | Accept |
|  | Training | 0.8 | 0.97 | Accept |
|  | Organizational events | 0.4 | 0.87 | Reject |
|  | Time of last accident | 0.6 | 1 | Reject |
| Environmental characteristics | Assistive devices available | 0.8 | 0.97 | Accept |
|  | Control and protection devices | 1 | 0.93 | Accept |
|  | Cold and hot weather | 0.8 | 0.93 | Accept |
|  | Ventilation system | 0.8 | 0.93 | Accept |
|  | Construction accident factors | 0.6 | 0.83 | Reject |
|  | Lightening system | 1 | 1 | Accept |
| Warehouse  capacities | Warehouse ceiling height | 0.8 | 1 | Accept |
|  | Number of shelves | 0.6 | 1 | Reject |
|  | The total volume of the warehouse | 0.8 | 1 | Accept |
|  | The number of floors per shelf | 0.8 | 1 | Accept |
|  | Vertical distance between floors | 0.6 | 0.97 | Reject |
|  | Distance between shelves | 0.6 | 0.97 | Reject |
|  | The width of the corridors | 0.8 | 1 | Accept |
|  | Length of corridors | 0.4 | 1 | Reject |
| Warehouse output | Season and month | 0.8 | 0.97 | Accept |
|  | Day | 0.8 | 0.97 | Accept |
|  | hour | 0.6 | 0.96 | Reject |
